# Phytophotodermatitis From Lime Margaritas on a Mexico Vacation

**DOI:** 10.7759/cureus.59674

**Published:** 2024-05-05

**Authors:** Brigitte L Cochran, Jennifer Jallo, Alexis Coican, Kala Hurst, Josie Sagasser, Melinda F Greenfield

**Affiliations:** 1 Osteopathic Medicine, Philadelphia College of Osteopathic Medicine, Moultrie, USA; 2 Family Medicine, Lincoln Memorial University-DeBusk College of Osteopathic Medicine, Harrogate, USA; 3 Dermatology, Orange Park Medical Center, Jacksonville, USA; 4 Dermatology, Case Western Reserve University, Cleveland, USA; 5 Medicine, University of South Florida, Tampa, USA; 6 Dermatology, Advanced Dermatology and Cosmetic Surgery, Ponte Vedra Beach, USA

**Keywords:** photodermatitis, plant biochemistry, furocoumarin, sun exposure, phytophotodermatitis

## Abstract

Phytophotodermatitis is a type of contact dermatitis that occurs upon skin exposure to certain plant chemicals, known as furocoumarins, along with simultaneous sun exposure. This case details a 34-year-old patient who presented to the office with an asymptomatic, irregularly shaped, and hyperpigmented patch located on the left inferior middle back that had been present since a recent beach vacation in Mexico. Upon gathering the history, clinicians should inquire about recent sunlight exposure while consuming and/or touching phytotoxic plant derivatives found in most citrus plants. The history should correspond with the skin examination findings and conclude that a cutaneous phytotoxic reaction had occurred when a lime margarita contacted the hand, which was subsequently rubbed onto the patient’s back. This case highlights the importance of both taking a thorough history and physical examination and being aware of the broad range of skin manifestations to prevent unnecessary treatment, such as topical corticosteroids, for other skin disorders (the irregular presentation of atopic dermatitis, allergic contact dermatitis, and dermatitis unspecified) or improperly suspected child abuse in younger patient presentations.

## Introduction

Phytophotodermatitis is a type of phototoxic dermatitis that results from the topical contact of certain plant chemicals with simultaneous sun exposure, particularly ultraviolet A (UVA) [1-4]. The chemical involved in this process derives from many plant families, most commonly Umbelliferae and Rutaceae [2,5]. The Umbelliferae family includes celery, wild parsnip, parsley, and hogweed, while Rutaceae includes citrus fruits, i.e., lime, lemon, grapefruit, and bitter and bergamot oranges [2,5]. The noteworthy compounds in each of these families are called furocoumarins [1]. Furocoumarins are made up of psoralen isomers, which typically remain as inactive chemicals unless exposed to UVA [4]. Once exposed, a photosensitivity reaction occurs, causing damage to cell DNA and membranes and subsequent cell destruction and injury to the epidermis [1]. This reaction causes a broad spectrum of cutaneous manifestations that begin as an acute phase and sometimes progress into a chronic phase [6,7]. The acute phase can be completely asymptomatic, or it can involve inflammatory patterns ranging from an erythematous or edematous rash to vesicles or bullae that are sometimes pruritic just hours to days after exposure [6,8]. These skin lesions can also present as either linear or bizarre, irregularly shaped patterns [5,6]. If the acute phase does not resolve spontaneously, then a chronic phase can occur due to a delayed post-inflammatory reaction by psoralen-stimulated melanin hyperproduction, leading to hyperpigmented skin lesions that can last weeks for months [8]. For this reason, early recognition is key to potentially preventing this progression from acute to chronic phase.

Given the wide range of skin manifestations, it is also important for clinicians to be aware of this potential diagnosis given its ability to resemble other dermatological conditions, including but not limited to allergic contact dermatitis, photoallergy, cellulitis, burns, or possible child abuse [2]. In establishing a diagnosis, a detailed history inquiring about previous photosensitivity reactions and recent exposure to psoralen-containing substances in sunlight is necessary [7,8]. Additionally, a physical examination should include a full skin examination including the scalp, head, neck, chest, abdomen, back, upper and lower extremities (including interdigital web spaces, fingernails, and toenails) [7]. If still unable to make a clear clinical diagnosis, some recommend photopatch testing (i.e., applying a miniscule amount of chemicals to the back and then exposure to ultraviolet {UV} light) to rule out other diagnoses such as allergic contact dermatitis or photoallergy, which, in contrast to phytophotodermatitis, are hypersensitivity reactions [7].

We present a case report of a female patient who was diagnosed with lime-induced phytophotodermatitis after a beach vacation in Mexico. A thorough history and physical examination led to this most likely diagnosis.

## Case presentation

This case details a 34-year-old Caucasian female who presented to the office as a new patient for an evaluation of a hyperpigmented patch located on the left inferior middle back in an irregular pattern. The patient stated that she noticed the area of discoloration shortly after a trip to Mexico. She denied any previous treatment of the area involved. She has no previous history of skin cancer, no family history of melanoma, and no family history of non-melanoma skin cancer.

A total body skin examination was performed. Palpations of the cervical lymph nodes did not reveal any lymphadenopathy. 

**Figure 1:**
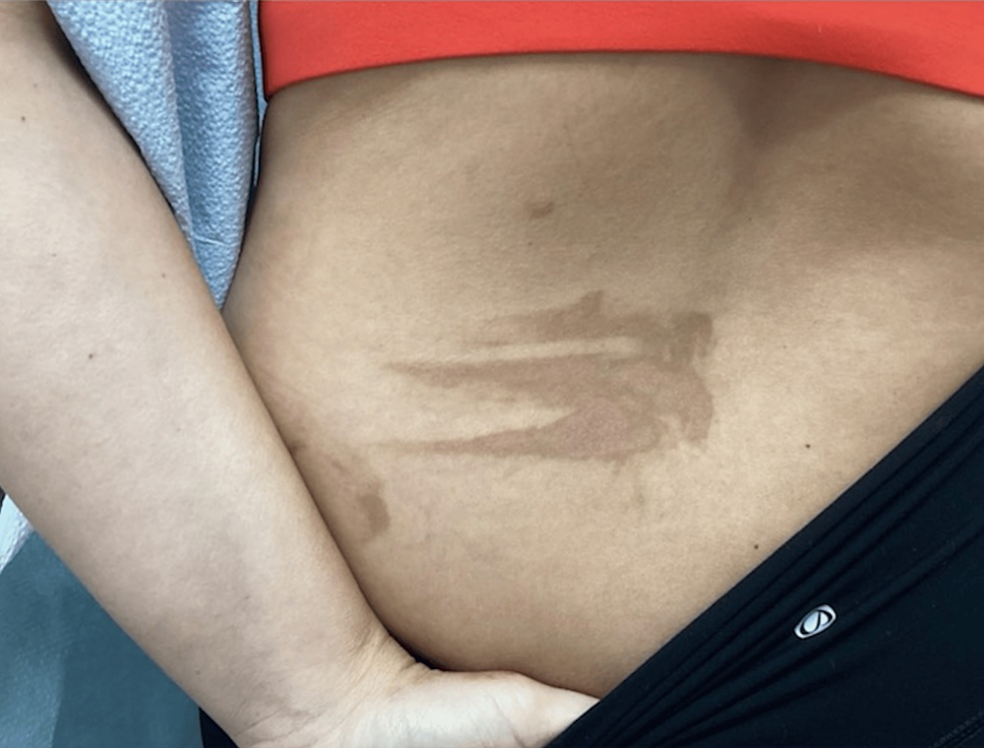
Clinical presentation of a three-striped hyperpigmented patch located on the left lower back, expanding into the left middle back. The patch has a streak located toward the lateral aspect giving clinical clues that ruled out idiopathic causes and narrowed toward self-induced phytophotodermatitis caused by the second, third, and fourth digits of the left hand touching the left medial back after being exposed to a photosensitizing agent such as a lime margarita that contained citrus.

An examination of the back revealed a hyperpigmented macular patch in an irregular pattern that gave clinical findings that indicated a non-idiopathic cause of the eruption of the lesion (Figure 1). Further questioning of the patient revealed that she had just returned from a beach vacation in Mexico. The patient was asked if she had any contact with exposure to limes or citrus, and the patient confirmed drinking lime margaritas. The conclusion was made that the patient may have touched her citrus drink and then subsequently brushed her left second, third, and fourth digits on her left inferior middle back and grazed her left hand laterally to cause the irregular pattern (Figure 1).

After the diagnosis of lime-induced phytophotodermatitis was determined, the patient was counseled to wash skin thoroughly after exposure to not only limes and citrus but also celery, figs, parsnip, and plants with psoralens (hogweed and Queen Anne’s lace). It was communicated to the patient that phytophotodermatitis is a benign finding and that it can persist for several weeks before it fully resolves and may leave irregular brown spots on the skin for months after exposure.

The patient was given triamcinolone acetonide 0.1% topical cream to apply to the affected area of the back twice a day for two weeks and then stop for one week and only use for flares of any possible future pruritic symptoms. The patient was educated that the corticosteroid cream will help decrease any active inflammation in the skin but likely will not remove the pigment. The patient was warned to avoid using any home remedies as this might exacerbate the hyperpigmentation. It was also discussed that if the phytophotodermatitis does not resolve within the following months, a bleaching cream of hydroquinone would be the next step. The patient was counseled on the importance of keeping the area out of the sun and applying sunscreen to the area, as further sun exposure would make the hyperpigmentation harder to eradicate.

## Discussion

The management of phytophotodermatitis involves abrupt contact cessation of the suspected offending agent. Begin with patient education and reassurance, emphasizing the benign nature of the disease process, and although aesthetically unpleasant, it usually resolves without long-term sequelae [7,8]. Discussing the expectations of the course of the disease is important. Next, patient counseling and education should be maximized. Patients should be told to prioritize sun-protective measures to prevent the condition from worsening. Patients should also be advised to avoid sun exposure and apply a minimum of sun protection factor (SPF) 30 whenever planning to spend any time outdoors [2]. If a concern to the patient, whether symptomatically or cosmetically, one can discuss further options. For symptomatic management in the acute phase, given that it is non-immunological and inflammatory, this can best be treated as if it was a sunburn, which involves cool compresses, emollients, and/or oral analgesics [1,2]. If pruritic or inflamed, physicians may prescribe topical, low-potency corticosteroids [6]. If widespread, you can attempt a 2-3-week course of corticosteroids [1]. Advise not to attempt topical anesthetics due to the potential of contact allergy. On the other hand, in the chronic phase, topical steroids are not typically indicated, being that the inflammatory process has already resolved [6]. As for cosmetic management, you can trial bleaching products, such as hydroquinone [9]. However, lasers and home remedies are generally not recommended as they do not have strong evidence for improvement in the skin condition [9]. One study suggested pulsed dye laser (PDL) as a potential treatment for post-inflammatory hyperpigmentation (PIH) in those with phototypes II and III; however, other skin types could be more prone to worsening hyperpigmentation [9].

It is important to distinguish the correct diagnosis when examining patients with this type of contact dermatitis. Other diagnoses that could be mistaken for phytophotodermatitis include the irregular presentation of atopic dermatitis (i.e., normally affects the flexural surfaces of the antecubital and popliteal fossa), allergic contact dermatitis, irritant contact dermatitis, photoallergy, cellulitis, burn, and possible child abuse in younger patient presentations [2]. A thorough history should be obtained by the physician such as inquiring about any recent beach vacations and/or any type of sunlight exposure after consuming liquid or solid phytotoxic plants such as citrus, celery, and parsnip [2].

Failing to recognize phytophotodermatitis could lead to unnecessary treatments such as long-term topical corticosteroids and antibiotics use, as well as oral antibiotic medication administration.

The limitations of this research include that a punch biopsy and/or photopatch testing was not performed to confirm the final diagnosis. Previous studies in rat subjects that were tested with lemon peel or lemon juice application to the shaved skin, which was then exposed to UV light, were evaluated for phytophotodermatitis by punch biopsy with histological analysis with hematoxylin and eosin staining [10]. These punch biopsies demonstrated the degeneration of the epithelium, which was similar to blistering skin conditions. It should be noted that this study, as well as a few other studies, analyzed phytophotodermatitis punch biopsy samples, which were indeterminate. Additionally, due to many clinicians opting to not perform punch biopsies on patients due to the confidence of the final diagnosis through both history and clinical examination findings, there is not enough clinical data regarding punch biopsy findings that indicate a diagnosis of phytophotodermatitis on dermatopathology examination [11,12]. Therefore, due to the combination of both specific history question answers (i.e., the patient’s recent beach vacation in Mexico, in which she was in contact with margaritas made with lime) and the clinical presentation, it was deemed unnecessary to pursue punch biopsy and/or photopatch testing.

## Conclusions

We report a case of a patient who acquired phytophotodermatitis following a beach vacation in Mexico in which subsequent sun exposure and plant chemicals produced a skin reaction. Given its purely clinical diagnosis from literature in the past, our case highlights the challenges in diagnosing phytophotodermatitis, which often mimics other dermatological conditions. Clinicians should be aware of the wide variety of skin manifestations in this process to avoid potential misdiagnoses and eventual unnecessary treatment modalities. The acute phase, the predominant stage of this disease process, typically resolves without intervention. However, it can occasionally produce local inflammatory-like symptoms, such as blisters or edema; then, treatment with topical, low-potency corticosteroids should be trialed. Additionally, only sometimes can this transform into a chronic phase. Otherwise, education on preventative measures, such as skin protection, remains paramount in avoiding future occurrences and unneeded medical interventions.
